# Exploring masculinities in rehabilitation research: A scoping review protocol

**DOI:** 10.1371/journal.pone.0361592

**Published:** 2026-10-30

**Authors:** Sing-Han Chiu, Janet Njelesani

**Affiliations:** Department of Occupational Therapy, Steinhardt School of Culture, Education, and Human Development, New York University, New York, New York, United States of America; Omeo Kumar Das Institute of Social Change and Development, INDIA

## Abstract

**Rationale:**

Rehabilitation involves help-seeking, engagement with professionals, adherence to treatment plans, adaptation to bodily changes, and participation in everyday life, all of which may be shaped by masculinities. Although masculinities have been examined in several health-related fields, existing literature on masculinities in rehabilitation remains fragmented across conditions, settings, and rehabilitation disciplines. To our knowledge, no review has mapped how masculinities have been conceptualized and examined across rehabilitation contexts.

**Objectives:**

This scoping review aims to map the nature and extent of peer-reviewed literature examining masculinities in rehabilitation contexts.

**Methods:**

This review will follow Arksey and O’Malley’s framework and Joanna Briggs Institute guidance and will be reported in accordance with the PRISMA Extension for Scoping Reviews. Searches were conducted in Scopus, Web of Science, PubMed, PsycINFO, CINAHL, ERIC, and Global Index Medicus. Key journals focused on men and masculinities and reference lists of included articles and relevant reviews will also be searched. Eligible sources will include English-language peer-reviewed empirical studies and conceptual or theoretical papers that examine masculinities in relation to rehabilitation services, experiences, practices, or contexts. Two reviewers will independently screen titles, abstracts, and full texts. Data will be charted in Google Sheets and analyzed using descriptive statistics and qualitative content analysis.

**Expected results:**

The review will provide a descriptive map of how masculinities have been conceptualized, operationalized, and examined across rehabilitation contexts.

**Conclusion:**

Findings will identify knowledge gaps and inform future rehabilitation research that more explicitly considers gendered processes.

## Introduction

Rehabilitation is a health strategy that aims to optimize functioning, reduce disability, and support people across the life course to participate in meaningful life roles [1]. The need for rehabilitation is substantial, with approximately 2.41 billion people worldwide living with health conditions that may benefit from rehabilitation [2]. Rehabilitation is concerned not only with using interventions to improve function but also with understanding how people with disabilities participate in their social and environmental contexts, so that interventions can be appropriately designed and implemented. Therefore, understanding rehabilitation requires attention to the social and cultural factors that shape people’s experiences of the rehabilitation process, including gender. However, a previous scoping review indicated that rehabilitation research often treats sex and gender as demographic variables, rather than as social processes that shape rehabilitation experiences [3]. This suggests the need to examine how gender may shape people’s access to, engagement with, and experiences of rehabilitation [4,5].

Examining gender in health and rehabilitation research also requires attention to masculinities, as men’s experiences are often studied without being analyzed as gendered experiences. Early feminist critiques challenged the androcentric tendency in health-related research to treat male experiences as the universal norm [6,7]. These critiques have importantly expanded attention to women’s experiences and to sexual and gender minority groups. However, men’s experiences are still not always examined as gendered experiences, particularly when men are treated as the unmarked or default group [8]. This is especially relevant for men who may hold a privileged position within the gender order while also experiencing marginalization through aging, illness, or disability [9,10]. Because older men, disabled men, and men living with chronic conditions are common populations in rehabilitation, masculinities provide an important perspective for examining how gender operates within rehabilitation contexts.

Masculinities refer to diverse meanings associated with being a man across different contexts, life stages, social positions, and theoretical paradigms [11]. This review uses the term masculinities rather than masculinity to emphasize that masculinities are not fixed or singular. Several paradigms have offered different ways of understanding masculinities. Across the literature, masculinities have been understood in different ways, including as traits associated with biological sex [12], as gender roles and norms learned through socialization [13,14], as identities shaped through psychological development [15], and as socially constructed practices organized through everyday interactions [16] and relations of power [17]. These perspectives highlight that masculinities are multiple and complex rather than universal or self-evident. This conceptual diversity matters for a scoping review because studies may define, operationalize, and examine masculinities in different ways across rehabilitation contexts.

Masculinities are relevant to rehabilitation because rehabilitation involves behaviors, interactions, and bodily experiences that may be shaped by gendered expectations. Courtenay [18] argued that health behaviors can function as social practices through which masculinities are demonstrated. In rehabilitation contexts, help-seeking, professional engagement, treatment adherence, and acceptance of support may therefore become ways through which masculinities are expressed, negotiated, or challenged. Existing reviews have shown that masculinities are linked to a range of health outcomes and behaviors relevant to rehabilitation. For instance, conformity to traditional masculine norms has been associated with poorer mental health outcomes [19], less favorable attitudes toward psychological help-seeking [19–21], and higher levels of adolescent internalizing problems [22]. However, these associations are not uniform. Depending on the context, masculinities may operate as either risk or protective factors. Research indicated that although masculinity constructs were generally associated with poorer health behaviors, some dimensions, such as emotional control and achievement orientation, could serve as protective factors in certain contexts [23]. Positive masculinity frameworks also argue for exploring how the positive potential of masculinities can improve health, rather than focusing only on toxic forms of masculinity [24]. A small body of rehabilitation research has examined masculinities in contexts such as traumatic brain injury rehabilitation, psychosocial rehabilitation, and cardiac rehabilitation [25–28]. These studies suggest that masculinities may shape rehabilitation-related behaviors and experiences, but current evidence remains dispersed across specific conditions and rehabilitation settings.

However, the existing literature on masculinities in rehabilitation remains fragmented across conditions, settings, and rehabilitation fields. Existing reviews on masculinities and health have largely focused on mental health, sexual health, partner violence, and gender-based violence. In rehabilitation-related literature, available reviews have focused on specific populations or conditions, such as men participating in cancer rehabilitation [29] or men with spinal cord injury [30]. Although these reviews provide important insights, to our knowledge, no review has mapped how masculinities have been conceptualized and examined across rehabilitation contexts. Therefore, this proposed scoping review aims to map the nature and extent of the existing literature that examines masculinities in rehabilitation contexts.

## Methods

### Study design

This scoping review will follow the methodological framework developed by Arksey and O’Malley [31], supplemented by the updated methodological guidance from the Joanna Briggs Institute (JBI) [32]. The review will involve five core stages: (1) identifying the research questions; (2) identifying relevant studies; (3) selecting eligible studies; (4) charting the data; and (5) collating, summarizing, and reporting the results. The completed review will be reported in accordance with the Preferred Reporting Items for Systematic Reviews and Meta-Analyses Extension for Scoping Reviews (PRISMA-ScR) guideline [33] to promote transparent and complete reporting.

This protocol has been registered on the Open Science Framework (OSF) and is currently under embargo. Any substantive amendments to this protocol will be documented and reported in the final published scoping review.

At the time of submission, this scoping review has not yet generated results. Database searches were conducted on June 10, 2026. We anticipate completing title and abstract screening by the end of July 2026, full-text screening by the end of August 2026, data charting by the end of September 2026, and analysis by the end of November 2026. This timeline is an estimate and may be adjusted as the review process progresses.

### Stage 1: Identifying the research question

This scoping review aims to answer the following primary question: What is known from the existing literature about masculinities in rehabilitation contexts? Specifically, the review will address the following secondary questions:

1. How are masculinities conceptualized, defined, and operationalized in literature on rehabilitation contexts?
2. What populations, health conditions, rehabilitation settings, professional disciplines, and geographical contexts have been represented in this literature?
3. To what extent and in what ways does the literature examine how masculinities shape rehabilitation processes, including how individuals access, enter, participate, continue, and leave rehabilitation services?
4. What knowledge gaps and underrepresented areas can be identified in the existing literature?

### Stage 2: Identifying relevant studies

Guided by the JBI guideline [32], this review uses the Population-Concept-Context (PCC) framework to refine our criteria and search strategies to identify relevant studies (Table 1).

**Table 1:** PCC framework: Inclusion and exclusion criteria.

Population	Individuals receiving, seeking, providing, or otherwise interacting with rehabilitation or rehabilitation-related services, including clients, family members, practitioners, and community partners, where applicable. Mixed-gender samples may be included when masculinities are explicitly examined.
Concept	Masculinities and related constructs, including masculine norms, manhood, male identity, gender role conflict, gender role strain, machismo, hegemonic masculinity, or closely related concepts.
Context	Rehabilitation and rehabilitation-related contexts, including occupational therapy, physical therapy, speech-language therapy, psychosocial rehabilitation, and other services with a clear rehabilitation focus.

The search strategy will include additional terms and synonyms related to masculinities and rehabilitation (e.g., gender role conflict and gender role strain) to ensure comprehensive retrieval of relevant literature.

#### Inclusion criteria.

Articles will be included if they: (1) are peer-reviewed journal articles published in English; (2) are empirical qualitative, quantitative, or mixed-methods studies, or theoretical or conceptual papers; (3) include individuals receiving, seeking, providing, or otherwise interacting with rehabilitation or rehabilitation-related services, including clients, family members, practitioners, or community partners, where applicable; (4) examine masculinities; and (5) are situated within the scope of rehabilitation. Studies will be considered rehabilitation-related when the authors explicitly position the study within rehabilitation or a predefined rehabilitation profession, and rehabilitation is substantively related to the study topic. Predefined rehabilitation professions include occupational therapy, physical therapy/physiotherapy, and speech-language therapy/pathology. Studies explicitly situated within rehabilitation programs or services, such as cardiac, cancer, neurological, psychosocial/psychiatric, or vocational rehabilitation, will also qualify. However, recruitment from a rehabilitation setting alone will not be sufficient if rehabilitation-related experiences, services, practices, or professional contexts are not substantively examined. Studies with mixed-gender samples will be included when masculinities are explicitly examined.

#### Exclusion criteria.

Articles will be excluded if they: (1) are grey literature, books, book chapters, dissertations, conference abstracts, unpublished studies, or reports; (2) are literature reviews, such as scoping review, systematic review, or meta-analysis; (3) refer to masculinities only in the background or discussion without examining them in the findings, analysis, or central argument in the theoretical papers; (4) focus on male participants or sex differences without examining masculinities; or (5) recruit participants with disabilities from rehabilitation settings without directly examining rehabilitation-related services, experiences, practices, or contexts. Studies will not be excluded solely because masculinities are not stated in the aim or research questions, provided that masculinity-related concepts are substantively examined in the findings or analysis.

#### Search strategy.

The scoping review search process is iterative [32]. The strategy has been refined through multiple discussions among members of the research team and a consultation with rehabilitation sciences librarians at New York University. The following sections describe the information sources, search terms, and search process.

To identify relevant databases, we reviewed systematic and scoping reviews examining masculinities in health and psychology-related fields and identified five commonly used databases: Scopus, Web of Science, PubMed, PsycINFO, and CINAHL. In consultation with the librarians, we added ERIC to broaden the coverage of education-related literature and Global Index Medicus to improve the identification of health research from regions and journals that may be underrepresented in major medical databases. In addition to database searching, we will also hand-search four journals with a strong focus on men and masculinities: *Men and Masculinities, American Journal of Men’s Health, Psychology of Men & Masculinities, and Journal of Men’s Health.* Lastly, relevant review articles identified during screening will be recorded, even though reviews are excluded from the final synthesis. These may include reviews addressing masculinities as well as broader reviews of men’s experiences or gender-related issues in rehabilitation. The reference lists of these reviews and included articles will be searched to identify potentially eligible studies that may not have been captured by the database search.

This supplementary search is intended to identify potentially eligible articles that substantively examine masculinities but do not mention masculinity-related terms in their abstracts.

After selecting the databases, we developed an initial search strategy and conducted preliminary searches to refine the search terms. The first preliminary search was conducted in PubMed on June 5, 2026. The initial search strategy included masculinity-related terms as well as broader terms referring to male populations, such as male, man, and boy. However, these broader terms retrieved many irrelevant articles that mentioned male participants only as part of the sample characteristics without examining masculinities. For example, none of the first ten most relevant records addressed masculinities. We therefore removed general male-related terms from the search strategy and retained terms that more specifically captured masculinities.

The rehabilitation-related search terms were also refined. The initial strategy listed multiple rehabilitation fields, including cardiac, cancer, musculoskeletal, community-based, and psychosocial rehabilitation. Following consultation with a librarian, we retained the core rehabilitation terms and selected terms related to the rehabilitation professions most relevant to the review. The revised PubMed search strategy was tested on June 10, 2026 (Table 2). The final search strategy for each database is provided in S1 Appendix.

**Table 2:** Initial and final PubMed search strategies.

Initial searching term	(Men [Mesh] OR “Gender Role” [Mesh] OR “Masculinity” [Mesh] OR Man [Title/Abstract] OR Men [Title/Abstract] OR Boy [Title/Abstract] OR Boys [Title/Abstract] OR masculin* [Title/Abstract] OR manhood [Title/Abstract] OR “male identit*” [Title/Abstract] OR machismo* [Title/Abstract] OR “gender role*” [Title/Abstract] OR “gender norm*” [Title/Abstract]) AND (“Rehabilitation”[Mesh] OR “Occupational Therapy”[Mesh] OR “Physical Therapy Modalities”[Mesh] OR “Exercise Therapy”[Mesh] OR “Cardiac Rehabilitation”[Mesh] OR “Neurological Rehabilitation”[Mesh] OR “Psychiatric Rehabilitation”[Mesh] OR “Rehabilitation of Speech and Language Disorders”[Mesh] OR “Rehabilitation, Vocational”[Mesh] OR “Telerehabilitation”[Mesh] OR rehab*[Title/Abstract] OR “occupational therap*”[Title/Abstract] OR “physical therap*”[Title/Abstract] OR physiotherap*[Title/Abstract] OR “exercise therapy”[Title/Abstract] OR “cardiac rehab*”[Title/Abstract] OR “cancer rehab*”[Title/Abstract] OR “neuro rehab*”[Title/Abstract] OR “musculoskeletal rehab*”[Title/Abstract] OR “vocational rehab*”[Title/Abstract] OR “community rehab*”[Title/Abstract] OR telerehabilitation[Title/Abstract])
Final searching term	(“Gender Role”[Mesh] OR “Masculinity”[Mesh] OR masculin*[Title/Abstract] OR manhood[Title/Abstract] OR “male identit*”[Title/Abstract] OR “male role norm*”[Title/Abstract] OR machismo*[Title/Abstract] OR “gender role conflict”[Title/Abstract] OR “gender role strain”[Title/Abstract] OR “gender role expectation*”[Title/Abstract] OR “gender norm*”[Title/Abstract]) AND (“Rehabilitation”[Mesh] OR “Occupational Therapy”[Mesh] OR “Physical Therapy Modalities”[Mesh] OR “Psychiatric Rehabilitation”[Mesh] OR rehab*[Title/Abstract] OR “occupational therap*”[Title/Abstract] OR “physical therap*”[Title/Abstract] OR physiotherap*[Title/Abstract])

### Stage 3: Study selection

Covidence will be used to import references, remove duplicates, and support the screening process [34]. The review team will include two researchers and two research assistants. Sing-Han Chiu (SHC) is a PhD student in Occupational Therapy, and Janet Njelesani (JN) is an Associate Professor in Occupational Therapy who has published multiple scoping reviews [35,36]. The research assistants have backgrounds in health-related fields, including psychology, sociology, and life sciences. SHC and the research assistants will conduct the screening. JN will serve as the third reviewer when disagreements cannot be resolved through discussion.

#### Pilot screening.

The eligibility criteria were pilot tested using a sample of 30 records during the title and abstract screening stage. Three reviewers independently screened the 30 records. The team then reviewed disagreements to determine whether (1) the inclusion and exclusion criteria are clearly stated, and (2) reviewers are interpreting the eligibility criteria consistently. The criteria will be refined if needed before formal screening begins.

#### Title and abstract review.

Each title and abstract will be independently reviewed by two reviewers using the inclusion and exclusion criteria. Records considered potentially relevant will proceed to full-text review. Disagreements will be discussed by the reviewers and, if unresolved, adjudicated by JN. At this stage, articles will not be excluded solely because masculinities or rehabilitation are not explicitly mentioned in the abstract, provided that the record otherwise suggests potential relevance. For masculinities, records focusing on men’s experiences or related gender concepts, such as gender roles, will be retained for full-text review. For rehabilitation, studies addressing broader processes such as functional recovery, adaptation to disability, assistive technology use, return to work, participation, illness experience, or disability will also be retained, even when rehabilitation or a rehabilitation profession is not explicitly mentioned. Such records will be marked as “maybe” and assessed in greater detail during full-text review. At full-text review, eligibility will be determined based on whether masculinities are substantively examined and whether the study is explicitly situated within rehabilitation services, practices, programs, or professions.

#### Full text review.

Each full-text article will be independently reviewed by two reviewers. Disagreements will be resolved through discussion or adjudicated by JN when necessary. Reasons for exclusion will be documented in Covidence. Covidence will automatically produce a PRISMA flow diagram, and the review will be reported in accordance with PRISMA-ScR [33].

#### Quality appraisal.

Critical appraisal is not required as the purpose of this scoping review is to map the scope of the existing literature and not identify methodological gaps.

### Stage 4: Charting the data

Google Sheets will be used to extract and organize the data. For all included articles, basic descriptive information will be extracted, including title, author(s), journal name, year of publication, country or region, publication type, and study design, where applicable. Additional data will be charted according to publication type and the review questions. For empirical research articles, extracted data will include population or stakeholder group, health condition, rehabilitation context, masculinity-related concepts or theoretical framework, how masculinities were defined or operationalized, rehabilitation-related processes or experiences examined, key findings related to masculinities and rehabilitation, and research gaps identified by the authors. For conceptual or theoretical papers, extracted data will include the rehabilitation issue addressed, masculinity-related concepts or theoretical framework, central argument, and implications or gaps identified for rehabilitation research or practice.

After full-text screening, the data-charting form will be pilot tested by two researchers using a sample of five included articles. The form will then be refined, and additional extraction fields may be developed based on the types of studies included in the review. At least two individuals will be involved in the data-charting process for each included record. One reviewer will read the full article and extract the relevant information, and a second reviewer will review the charted data for accuracy and completeness. Any disagreements will be resolved through discussion. If consensus cannot be reached, a senior researcher (JN) will be consulted. Data charting will remain an iterative process, and relevant fields may be added or refined if useful information emerges during extraction. Any additions, removals, or revisions to the data-charting framework will be documented in a shared log, including the date, decision, and rationale. Important post-protocol changes will be reported in the final scoping review.

### Stage 5: Collating, summarizing, and reporting the results

Following JBI recommendations for the extraction, analysis, and presentation of results in scoping reviews, this review aims to provide a descriptive map of the existing literature related to masculinities in rehabilitation contexts [37]. First, descriptive characteristics of the included sources of evidence will be summarized using frequency counts and percentages. These characteristics may include publication year, study design, population, health condition, rehabilitation setting, professional discipline, geographical context, and other relevant variables identified through the data-charting process. The specific variables to be analyzed will be determined based on the finalized data-charting form developed in Stage 4. Results will be presented using tables, figures, or other graphical displays to facilitate the mapping of the evidence base.

Consistent with JBI guidance, this review will not employ thematic synthesis, meta-aggregation, or other interpretive qualitative synthesis approaches, as these methods are intended to generate interpretations and therefore extend beyond the descriptive purpose of scoping reviews [37]. Instead, qualitative content analysis will be used to organize and categorize data extracted from the included articles [38]. The review team will first read and reread the extracted data to become familiar with the content. Initial codes, category definitions, and decision rules will then be developed and pilot tested using a sample of five included articles. Three reviewers will independently code the pilot sample and meet to compare coding decisions, resolve discrepancies, and refine the codebook. The refined codebook will then be applied to the remaining included studies, with further revisions made through team discussion when needed. Regular peer-debriefing meetings will be held to support the trustworthiness of the analytic process. An audit trail will be maintained to document coding decisions, revisions to the codebook, disagreements, and how disagreements are resolved [39]. The resulting categories will be used to descriptively map how masculinities have been examined across rehabilitation contexts.

#### Presentation of results.

Data regarding the search and inclusion of articles will be reported in line with PRISMA-ScR reporting guidelines, including a PRISMA-ScR flow diagram. This scoping review will include a descriptive summary of the included literature, presented in tables and figures as appropriate. The results will be organized into descriptive categories aligned with the review questions. The emphasis will be on mapping how masculinities have been conceptualized, operationalized, examined, and discussed across rehabilitation-related contexts.

## Limitations

This review has several limitations. First, because the search strategy focuses on explicit masculinity-related terms, the review may miss articles that examine masculinities in rehabilitation contexts without using masculinity-related terms in the title and abstract. Some studies may not be designed with masculinities as their primary focus but may still include relevant findings in their results. To reduce this risk, reference list searching will be conducted for included articles and relevant reviews to identify potentially relevant studies. However, this approach may still be insufficient, particularly for recently published articles.

Second, some rehabilitation-related studies may be missed if they do not explicitly identify themselves as rehabilitation research or use rehabilitation-related terminology. For example, studies may examine functional recovery, adaptation to disability, assistive technology use, return to work, or participation after illness or injury without using the term rehabilitation. Although the search strategy includes core rehabilitation terms and professional disciplines, the boundary of rehabilitation remains complex, and some relevant studies may not be captured.

Third, this review will include only peer-reviewed articles published in English. Grey literature, books, book chapters, unpublished studies, dissertations, conference abstracts, and reports will be excluded. As a result, the review may miss relevant evidence published in other languages or presented in other types of sources.

## Supporting information

S1 Appendix This is the search strategies for each database. (DOCX)

S2 Appendix This is the PRISMA-P Checklist. (DOCX)
