# Supplementary material for: Exploring masculinities in rehabilitation research: A scoping review protocol
Source: PLoS One. 2026 Oct 30;21(10):e0361592. doi: 10.1371/journal.pone.0361592 (PMC13722176; doi:10.1371/journal.pone.0361592)
Supplement: Supplementary file 1 [file pone.0361592.s001.docx]

# Appendix 1: S1_search strategies for each database

# Searching process for PubMed

|  | Search term(s) | Results |
| --- | --- | --- |
| #1 | Men [Mesh] OR "Gender Role" [Mesh] OR "Masculinity" [Mesh] OR Man [Title/Abstract] OR Men [Title/Abstract] OR Boy [Title/Abstract] OR Boys [Title/Abstract] OR masculin* [Title/Abstract] OR manhood [Title/Abstract] OR "male identit*" [Title/Abstract] OR machismo* [Title/Abstract] OR "gender role*" [Title/Abstract] OR "gender norm*" [Title/Abstract] | 1,229,373 |
| #2 | "Rehabilitation"[Mesh] OR "Occupational Therapy"[Mesh] OR "Physical Therapy Modalities"[Mesh] OR "Exercise Therapy"[Mesh] OR "Cardiac Rehabilitation"[Mesh] OR "Neurological Rehabilitation"[Mesh] OR "Psychiatric Rehabilitation"[Mesh] OR "Rehabilitation of Speech and Language Disorders"[Mesh] OR "Rehabilitation, Vocational"[Mesh] OR "Telerehabilitation"[Mesh] OR rehab*[Title/Abstract] OR "occupational therap*"[Title/Abstract] OR "physical therap*"[Title/Abstract] OR physiotherap*[Title/Abstract] OR "exercise therapy"[Title/Abstract] OR "cardiac rehab*"[Title/Abstract] OR "cancer rehab*"[Title/Abstract] OR "neuro rehab*"[Title/Abstract] OR "musculoskeletal rehab*"[Title/Abstract] OR "vocational rehab*"[Title/Abstract] OR "community rehab*"[Title/Abstract] OR telerehabilitation[Title/Abstract] | 653,451 |
| #3 | #1 AND #2 | 32,717 |
| #4 | "Gender Role"[Mesh] OR "Masculinity"[Mesh] OR masculin*[Title/Abstract] OR manhood[Title/Abstract] OR "male identit*"[Title/Abstract] OR "male role norm*"[Title/Abstract] OR machismo*[Title/Abstract] OR "gender role conflict"[Title/Abstract] OR "gender role strain"[Title/Abstract] OR "gender role expectation*"[Title/Abstract] OR "gender norm*"[Title/Abstract] | 16861 |
| #5 | #4 AND #2 | 212 |
| #6 | "Rehabilitation"[Mesh] OR "Occupational Therapy"[Mesh] OR "Physical Therapy Modalities"[Mesh] OR "Psychiatric Rehabilitation"[Mesh] OR rehab*[Title/Abstract] OR "occupational therap*"[Title/Abstract] OR "physical therap*"[Title/Abstract] OR physiotherap*[Title/Abstract] | 651,109 |
| #7 | #4 AND #6 | 212 |

# Final Search Strategies for Each Databases

| Database | Result | Searching term | Time |
| --- | --- | --- | --- |
| Web of Science | 372 | TS=(masculin* OR manhood OR "male identit*" OR "male role norm*" OR machismo* OR "gender role conflict" OR "gender role strain" OR "gender role expectation*" OR "gender norm*")  AND  TS=(rehab* OR "occupational therap*" OR "physical therap*" OR physiotherap*) | June 10, 2026 |
| Scopus | 539 | TITLE-ABS-KEY ( masculin* OR manhood OR "male identit*" OR "male role norm*" OR machismo* OR "gender role*" OR "gender norm*" OR "gender role conflict" OR "gender role strain" OR "gender role expectation*" )  AND  TITLE-ABS-KEY ( rehab* OR "occupational therap*" OR "physical therap*" OR physiotherap* ) | June 10, 2026 |
| CINAHL | 406 | (MH "Masculinity" OR MH "Gender Role" OR XB (masculin* OR manhood OR "male identit*" OR "male role norm*" OR machismo* OR "gender role conflict" OR "gender role strain" OR "gender role expectation*" OR "gender norm*"))  AND  (MH "Rehabilitation" OR MH "Occupational Therapy" OR MH "Physical Therapy" OR MH "Psychiatric Rehabilitation" OR XB ( rehab* OR "occupational therap*" OR "physical therap*" OR physiotherap* )) | June 10, 2026 |
| PubMed | 212 | ("Gender Role"[Mesh] OR "Masculinity"[Mesh] OR masculin* [Title/Abstract] OR manhood[Title/Abstract] OR "male identit*" [Title/Abstract] OR "male role norm*"[Title/Abstract] OR machismo* [Title/Abstract] OR "gender role conflict"[Title/Abstract] OR "gender role strain"[Title/Abstract] OR "gender role expectation*" [Title/Abstract] OR "gender norm*"[Title/Abstract])  AND  ("Rehabilitation"[Mesh] OR "Occupational Therapy"[Mesh] OR "Physical Therapy Modalities"[Mesh] OR "Psychiatric Rehabilitation" [Mesh] OR rehab*[Title/Abstract] OR "occupational therap*" [Title/Abstract] OR "physical therap*"[Title/Abstract] OR physiotherap*[Title/Abstract]) | June 10, 2026 |
| PsycINFO | 168 | ((masculin* or manhood or "male identit*" or "male role norm*" or machismo* or "gender role conflict" or "gender role strain" or "gender role expectation*" or "gender norm*")  and  (rehab* or "occupational therap*" or "physical therap*" or physiotherap*)).ti,ab. | June 10, 2026 |
| ERIC | 17 | ("masculinity" OR "masculine" OR "masculinities" OR "manhood" OR "male identity" OR "male role norm" OR "machismo" OR "gender role conflict" OR "gender role strain" OR "gender role expectation" OR "gender norm")  AND  ("rehabilitation" OR "rehab" OR "occupational therapy" OR "physical therapy" OR "physiotherapy") | June 10, 2026 |
| Global Index Medicus | 28 | ( MH:"Masculinity" OR TW:( masculinit* OR manhood* OR "male identity" OR "male identities" OR "male role norm" OR "male role norms" OR machismo* OR "gender role conflict" OR "gender role strain" OR "gender role expectation" OR "gender role expectations" OR "gender norm" OR "gender norms" ) )  AND   ( MH:( "Rehabilitation" OR "Occupational Therapy" OR "Physical Therapy Modalities" OR "Psychiatric Rehabilitation" ) OR TW:( rehab* OR "occupational therapy" OR "physical therapy" OR physiotherap* ) ) | June 10, 2026 |
