# Supplementary material for: Exploring masculinities in rehabilitation research: A scoping review protocol
Source: PLoS One. 2026 Oct 30;21(10):e0361592. doi: 10.1371/journal.pone.0361592 (PMC13722176; doi:10.1371/journal.pone.0361592)
Supplement: Supplementary file 3 [file pone.0361592.s003.docx]

**Manuscript Number: PONE-D-26-31876**

Exploring masculinities in rehabilitation research: A scoping review protocol

# Reviewer #1:

This is a well-conceived and clearly written scoping review protocol addressing an important and relatively underexplored intersection between masculinities and rehabilitation research. The rationale is convincing, and the primary and secondary research questions are clearly articulated and appropriate for a scoping review. The use of the Arksey and O’Malley framework, JBI guidance, and PRISMA-ScR provides a sound methodological foundation.

The protocol is generally detailed and reproducible. I particularly appreciate the use of independent screening, pilot testing of the eligibility criteria, procedures for resolving disagreements, pilot testing of the data-charting form, and the planned audit trail for the qualitative content analysis.

I have a few minor comments that may further strengthen the protocol:

1. Definition and boundaries of rehabilitation: The authors appropriately acknowledge that rehabilitation is a broad and sometimes difficult-to-delimit concept. It would be helpful to provide a slightly more explicit operational rule for determining whether studies addressing areas such as recovery, adaptation to disability, assistive technology, or return to work qualify as “rehabilitation-related” when rehabilitation terminology is not explicitly used. This would further improve consistency during screening.

**Our response to comment 1:**

Thank you for this helpful comment. We agree that the broad boundaries of rehabilitation require a clearer operational rule. We clarified that final eligibility will be based on both explicit positioning within rehabilitation and substantive relevance to rehabilitation. Studies will qualify when the authors explicitly situate the work within rehabilitation, a predefined rehabilitation profession, or an explicitly identified rehabilitation program or service, and rehabilitation is substantively related to the study topic. Recruitment from a rehabilitation setting alone will not be sufficient. For broader topics such as recovery, adaptation to disability, assistive technology use, return to work, or participation, potentially relevant records will be retained during title and abstract screening and evaluated at full-text review using these criteria. We have added these operational rules to the eligibility criteria and study-selection sections of the manuscript.

(paragraph 1, page 8)

…(4) examine masculinities; and (5) are situated within the scope of rehabilitation. Studies will be considered rehabilitation-related when the authors explicitly position the study within rehabilitation or a predefined rehabilitation profession, and rehabilitation is substantively related to the study topic. Predefined rehabilitation professions include occupational therapy, physical therapy/physiotherapy, and speech-language therapy/pathology. Studies explicitly situated within rehabilitation programs or services, such as cardiac, cancer, neurological, psychosocial/psychiatric, or vocational rehabilitation, will also qualify. However, recruitment from a rehabilitation setting alone will not be sufficient if rehabilitation-related experiences, services, practices, or professional contexts are not substantively examined. Studies with mixed-gender …

(paragraph 1, page 12)

…suggests potential relevance. For masculinities, records focusing on men’s experiences or related gender concepts, such as gender roles, will be retained for full-text review. For rehabilitation, studies addressing broader processes such as functional recovery, adaptation to disability, assistive technology use, return to work, participation, illness experience, or disability will also be retained, even when rehabilitation or a rehabilitation profession is not explicitly mentioned. Such records will be marked as “maybe” and assessed in greater detail during full-text review. At full-text review, eligibility will be determined based on whether masculinities are substantively examined and whether the study is explicitly situated within rehabilitation services, practices, programs, or professions.

2. Search sensitivity: The decision to remove broad terms such as “men,” “male,” and “boys” is understandable given the large number of irrelevant records retrieved during preliminary searching. However, this decision may increase the possibility of missing studies in which masculinity is substantively analyzed but not mentioned in the title or abstract. The supplementary hand-searching and reference-list searching partly address this issue. The authors may wish to clarify whether citation searching or another supplementary strategy will be used to further mitigate this limitation.

**Our response to comment 2:**

Thank you for raising this concern. We agree that removing broad population terms may increase the risk of missing relevant studies. Our original protocol already included reference-list searching of relevant reviews as a supplementary strategy. We have now clarified that these reviews are not limited to those explicitly focused on masculinities, but may also include reviews of men’s experiences or gender-related issues in rehabilitation contexts, such as cancer rehabilitation, prolonged disability, and assistive technology. Their reference lists will be searched to identify potentially eligible studies not captured by the database search. We have clarified this strategy in the revised manuscript.

(paragraph 2, page 9)

*…* Lastly, relevant review articles identified during screening will be recorded, even though reviews are excluded from the final synthesis. These may include reviews addressing masculinities as well as broader reviews of men’s experiences or gender-related issues in rehabilitation. The reference lists of these reviews and included articles will be searched to identify potentially eligible studies that may not have been captured by the database search.

(paragraph 3, page 15)

To reduce this risk, reference list searching will be conducted for included articles and relevant reviews to identify potentially relevant studies…

3. Data charting and analysis: The protocol appropriately describes data charting as iterative. However, because additional extraction fields and analytic variables may be added after reviewing included studies, I suggest briefly clarifying how substantive post-protocol changes to the charting framework or coding decisions will be documented. This would further strengthen transparency and reproducibility.

**Our response to comment 3:**

Thank you for this helpful suggestion. We maintain a shared decision log to document key methodological and operational decisions throughout the review. The log records the date, issue, decision, and rationale and is accessible to all review team members. We have clarified that substantive changes to the data-charting framework will be documented in this log and reported in the final scoping review. Coding decisions and revisions to the codebook will continue to be documented through the audit trail described in the analysis section.

(paragraph 2, page 13)

…Data charting will remain an iterative process, and relevant fields may be added or refined if useful information emerges during extraction. Any additions, removals, or revisions to the data-charting framework will be documented in a shared log, including the date, decision, and rationale. Important post-protocol changes will be reported in the final scoping review.

4. Minor editorial point: Please correct the spelling of “Rehablitation” in the keywords to “Rehabilitation” and conduct a final proofread for minor typographical issues.

**Our response to comment 4:**

Thank you for noting this. We corrected the spelling of “Rehabilitation” in the submission system and conducted a final proofread of the manuscript for minor typographical issues.
