# Supplementary material for: Exploring masculinities in rehabilitation research: A scoping review protocol
Source: PLoS One. 2026 Oct 30;21(10):e0361592. doi: 10.1371/journal.pone.0361592 (PMC13722176; doi:10.1371/journal.pone.0361592)
Supplement: Peer review history

# Peer review: Decision Letter 0

**DOI:** 10.1371/journal.pone.0361592.r001

**Authors:** Nitish Gogoi

24 Sep 2026

Dear Dr. Chiu,

Thank you for submitting your manuscript to PLOS One. After careful consideration, we feel that it has merit but does not fully meet PLOS One’s publication criteria as it currently stands. Therefore, we invite you to submit a revised version of the manuscript that addresses the points raised during the review process.

==============================

**ACADEMIC EDITOR'S COMMENT:**  Dear author, we have now received reviewers’ comments on your paper. Please revise your paper as suggested by the reviewer. Looking forward to receiving your paper.

==============================

Please submit your revised manuscript by Nov 22 2026 11:59PM. If you will need more time than this to complete your revisions, please reply to this message or contact the journal office at plosone@plos.org. When you're ready to submit your revision, log on to https://www.editorialmanager.com/pone/ and select the 'Submissions Needing Revision' folder to locate your manuscript file.

- A letter that responds to each point raised by the academic editor and reviewer(s). You should upload this letter as a separate file labeled 'Response to Reviewers'.
- A marked-up copy of your manuscript that highlights changes made to the original version. You should upload this as a separate file labeled 'Revised Manuscript with Track Changes'.
- An unmarked version of your revised paper without tracked changes. You should upload this as a separate file labeled 'Manuscript'.

If you would like to make changes to your financial disclosure, please include your updated statement in your cover letter. Guidelines for resubmitting your figure files are available below the reviewer comments at the end of this letter.

If applicable, we recommend that you deposit your laboratory protocols in protocols.io to enhance the reproducibility of your results. Protocols.io assigns your protocol its own identifier (DOI) so that it can be cited independently in the future. For instructions see: https://journals.plos.org/plosone/s/submission-guidelines#loc-laboratory-protocols. Additionally, PLOS One offers an option for publishing peer-reviewed Lab Protocol articles, which describe protocols hosted on protocols.io. Read more information on sharing protocols at https://plos.org/protocols?utm_medium=editorial-email&utm_source=authorletters&utm_campaign=protocols.

As the corresponding author, your ORCID iD is verified in the submission system and will appear in the published article. PLOS supports the use of ORCID, and we encourage all coauthors to register for an ORCID iD and use it as well. Please encourage your coauthors to verify their ORCID iD within the submission system before final acceptance, as unverified ORCID iDs will not appear in the published article. *Only*  the individual author can complete the verification step; PLOS staff *cannot*  verify ORCID iDs on behalf of authors.

We look forward to receiving your revised manuscript.

Kind regards,

Nitish Gogoi, Ph.D.

Academic Editor

PLOS One

Journal Requirements:

When submitting your revision, we need you to address these additional requirements.

1. Please ensure that your manuscript meets PLOS ONE's style requirements, including those for file naming. The PLOS ONE style templates can be found at https://journals.plos.org/plosone/s/file?id=wjVg/PLOSOne_formatting_sample_main_body.pdf and https://journals.plos.org/plosone/s/file?id=ba62/PLOSOne_formatting_sample_title_authors_affiliations.pdf

If the reviewer comments include a recommendation to cite specific previously published works, please review and evaluate these publications to determine whether they are relevant and should be cited. There is no requirement to cite these works unless the editor has indicated otherwise.

[Note: HTML markup is below. Please do not edit.]

Reviewers' comments:

Reviewer's Responses to Questions

**Comments to the Author**

1. Does the manuscript provide a valid rationale for the proposed study, with clearly identified and justified research questions?

Reviewer #1: Yes

**********

2. Is the protocol technically sound and planned in a manner that will lead to a meaningful outcome and allow testing the stated hypotheses?

Reviewer #1: Yes

**********

3. Is the methodology feasible and described in sufficient detail to allow the work to be replicable?

Reviewer #1: Yes

**********

4. Have the authors described where all data underlying the findings will be made available when the study is complete?

The PLOS Data policy

Reviewer #1: Yes

**********

5. Is the manuscript presented in an intelligible fashion and written in standard English?

Reviewer #1: Yes

**********

Please use the space provided to explain your answers to the questions above and, if applicable, provide comments about issues authors must address before this protocol can be accepted for publication. You may also include additional comments for the author, including concerns about research or publication ethics.

You may also provide optional suggestions and comments to authors that they might find helpful in planning their study.

Reviewer #1: This is a well-conceived and clearly written scoping review protocol addressing an important and relatively underexplored intersection between masculinities and rehabilitation research. The rationale is convincing, and the primary and secondary research questions are clearly articulated and appropriate for a scoping review. The use of the Arksey and O’Malley framework, JBI guidance, and PRISMA-ScR provides a sound methodological foundation.

The protocol is generally detailed and reproducible. I particularly appreciate the use of independent screening, pilot testing of the eligibility criteria, procedures for resolving disagreements, pilot testing of the data-charting form, and the planned audit trail for the qualitative content analysis.

I have a few minor comments that may further strengthen the protocol:

Definition and boundaries of rehabilitation: The authors appropriately acknowledge that rehabilitation is a broad and sometimes difficult-to-delimit concept. It would be helpful to provide a slightly more explicit operational rule for determining whether studies addressing areas such as recovery, adaptation to disability, assistive technology, or return to work qualify as “rehabilitation-related” when rehabilitation terminology is not explicitly used. This would further improve consistency during screening.

Search sensitivity: The decision to remove broad terms such as “men,” “male,” and “boys” is understandable given the large number of irrelevant records retrieved during preliminary searching. However, this decision may increase the possibility of missing studies in which masculinity is substantively analyzed but not mentioned in the title or abstract. The supplementary hand-searching and reference-list searching partly address this issue. The authors may wish to clarify whether citation searching or another supplementary strategy will be used to further mitigate this limitation.

Data charting and analysis: The protocol appropriately describes data charting as iterative. However, because additional extraction fields and analytic variables may be added after reviewing included studies, I suggest briefly clarifying how substantive post-protocol changes to the charting framework or coding decisions will be documented. This would further strengthen transparency and reproducibility.

Minor editorial point: Please correct the spelling of “Rehablitation” in the keywords to “Rehabilitation” and conduct a final proofread for minor typographical issues.

Overall, this is a strong and carefully developed protocol. The proposed review has the potential to provide a useful map of how masculinities have been conceptualized and examined across rehabilitation contexts. The comments above are primarily clarifications and minor methodological refinements rather than concerns requiring substantial redesign.

**********

what does this mean?). If published, this will include your full peer review and any attached files.

If you choose “no”, your identity will remain anonymous but your review may still be made public.

**Do you want your identity to be public for this peer review?**  For information about this choice, including consent withdrawal, please see our Privacy Policy

Reviewer #1: No

**********

[NOTE: If reviewer comments were submitted as an attachment file, they will be attached to this email and accessible via the submission site. Please log into your account, locate the manuscript record, and check for the action link "View Attachments". If this link does not appear, there are no attachment files.]

To ensure your figures meet our technical requirements, please review our figure guidelines: https://journals.plos.org/plosone/s/figures

You may also use PLOS’s free figure tool, NAAS, to help you prepare publication quality figures: https://journals.plos.org/plosone/s/figures#loc-tools-for-figure-preparation.

NAAS will assess whether your figures meet our technical requirements by comparing each figure against our figure specifications.

# Author comment: Author response to Decision Letter 1

**DOI:** 10.1371/journal.pone.0361592.r002

30 Sep 2026

Manuscript Number: PONE-D-26-31876

Exploring masculinities in rehabilitation research: A scoping review protocol

Reviewer #1:

This is a well-conceived and clearly written scoping review protocol addressing an important and relatively underexplored intersection between masculinities and rehabilitation research. The rationale is convincing, and the primary and secondary research questions are clearly articulated and appropriate for a scoping review. The use of the Arksey and O’Malley framework, JBI guidance, and PRISMA-ScR provides a sound methodological foundation.

The protocol is generally detailed and reproducible. I particularly appreciate the use of independent screening, pilot testing of the eligibility criteria, procedures for resolving disagreements, pilot testing of the data-charting form, and the planned audit trail for the qualitative content analysis.

I have a few minor comments that may further strengthen the protocol:

1. Definition and boundaries of rehabilitation: The authors appropriately acknowledge that rehabilitation is a broad and sometimes difficult-to-delimit concept. It would be helpful to provide a slightly more explicit operational rule for determining whether studies addressing areas such as recovery, adaptation to disability, assistive technology, or return to work qualify as “rehabilitation-related” when rehabilitation terminology is not explicitly used. This would further improve consistency during screening.

Our response to comment 1:

Thank you for this helpful comment. We agree that the broad boundaries of rehabilitation require a clearer operational rule. We clarified that final eligibility will be based on both explicit positioning within rehabilitation and substantive relevance to rehabilitation. Studies will qualify when the authors explicitly situate the work within rehabilitation, a predefined rehabilitation profession, or an explicitly identified rehabilitation program or service, and rehabilitation is substantively related to the study topic. Recruitment from a rehabilitation setting alone will not be sufficient. For broader topics such as recovery, adaptation to disability, assistive technology use, return to work, or participation, potentially relevant records will be retained during title and abstract screening and evaluated at full-text review using these criteria. We have added these operational rules to the eligibility criteria and study-selection sections of the manuscript.

(paragraph 1, page 8)

…(4) examine masculinities; and (5) are situated within the scope of rehabilitation. Studies will be considered rehabilitation-related when the authors explicitly position the study within rehabilitation or a predefined rehabilitation profession, and rehabilitation is substantively related to the study topic. Predefined rehabilitation professions include occupational therapy, physical therapy/physiotherapy, and speech-language therapy/pathology. Studies explicitly situated within rehabilitation programs or services, such as cardiac, cancer, neurological, psychosocial/psychiatric, or vocational rehabilitation, will also qualify. However, recruitment from a rehabilitation setting alone will not be sufficient if rehabilitation-related experiences, services, practices, or professional contexts are not substantively examined. Studies with mixed-gender …

(paragraph 1, page 12)

…suggests potential relevance. For masculinities, records focusing on men’s experiences or related gender concepts, such as gender roles, will be retained for full-text review. For rehabilitation, studies addressing broader processes such as functional recovery, adaptation to disability, assistive technology use, return to work, participation, illness experience, or disability will also be retained, even when rehabilitation or a rehabilitation profession is not explicitly mentioned. Such records will be marked as “maybe” and assessed in greater detail during full-text review. At full-text review, eligibility will be determined based on whether masculinities are substantively examined and whether the study is explicitly situated within rehabilitation services, practices, programs, or professions.

2. Search sensitivity: The decision to remove broad terms such as “men,” “male,” and “boys” is understandable given the large number of irrelevant records retrieved during preliminary searching. However, this decision may increase the possibility of missing studies in which masculinity is substantively analyzed but not mentioned in the title or abstract. The supplementary hand-searching and reference-list searching partly address this issue. The authors may wish to clarify whether citation searching or another supplementary strategy will be used to further mitigate this limitation.

Our response to comment 2:

Thank you for raising this concern. We agree that removing broad population terms may increase the risk of missing relevant studies. Our original protocol already included reference-list searching of relevant reviews as a supplementary strategy. We have now clarified that these reviews are not limited to those explicitly focused on masculinities, but may also include reviews of men’s experiences or gender-related issues in rehabilitation contexts, such as cancer rehabilitation, prolonged disability, and assistive technology. Their reference lists will be searched to identify potentially eligible studies not captured by the database search. We have clarified this strategy in the revised manuscript.

(paragraph 2, page 9)

… Lastly, relevant review articles identified during screening will be recorded, even though reviews are excluded from the final synthesis. These may include reviews addressing masculinities as well as broader reviews of men’s experiences or gender-related issues in rehabilitation. The reference lists of these reviews and included articles will be searched to identify potentially eligible studies that may not have been captured by the database search.

(paragraph 3, page 15)

To reduce this risk, reference list searching will be conducted for included articles and relevant reviews to identify potentially relevant studies…

3. Data charting and analysis: The protocol appropriately describes data charting as iterative. However, because additional extraction fields and analytic variables may be added after reviewing included studies, I suggest briefly clarifying how substantive post-protocol changes to the charting framework or coding decisions will be documented. This would further strengthen transparency and reproducibility.

Our response to comment 3:

Thank you for this helpful suggestion. We maintain a shared decision log to document key methodological and operational decisions throughout the review. The log records the date, issue, decision, and rationale and is accessible to all review team members. We have clarified that substantive changes to the data-charting framework will be documented in this log and reported in the final scoping review. Coding decisions and revisions to the codebook will continue to be documented through the audit trail described in the analysis section.

(paragraph 2, page 13)

…Data charting will remain an iterative process, and relevant fields may be added or refined if useful information emerges during extraction. Any additions, removals, or revisions to the data-charting framework will be documented in a shared log, including the date, decision, and rationale. Important post-protocol changes will be reported in the final scoping review.

4. Minor editorial point: Please correct the spelling of “Rehablitation” in the keywords to “Rehabilitation” and conduct a final proofread for minor typographical issues.

Our response to comment 4:

Thank you for noting this. We corrected the spelling of “Rehabilitation” in the submission system and conducted a final proofread of the manuscript for minor typographical issues.

Attachment Submitted filename: 00_Response_to_Reviewer_PLOS_ONE_R1_20260928.docx

# Editor report: Decision Letter 1

**DOI:** 10.1371/journal.pone.0361592.r003

**Authors:** Nitish Gogoi

13 Oct 2026

Exploring masculinities in rehabilitation research: A scoping review protocol

PONE-D-26-31876R1

Dear Dr. Chiu,

We’re pleased to inform you that your manuscript has been judged scientifically suitable for publication and will be formally accepted for publication once it meets all outstanding technical requirements.

Within one week, you’ll receive an e-mail detailing the required amendments. When these have been addressed, you’ll receive a formal acceptance letter and your manuscript will be scheduled for publication.

An invoice will be generated when your article is formally accepted. Please note, if your institution has a publishing partnership with PLOS and your article meets the relevant criteria, all or part of your publication costs will be covered. Please make sure your user information is up-to-date by logging into Editorial Manager at Editorial Manager® and clicking the ‘Update My Information' link at the top of the page. For questions related to billing, please contact billing support.

If your institution or institutions have a press office, please notify them about your upcoming paper to help maximize its impact. If they’ll be preparing press materials, please inform our press team as soon as possible -- no later than 48 hours after receiving the formal acceptance. Your manuscript will remain under strict press embargo until 2 pm Eastern Time on the date of publication. For more information, please contact onepress@plos.org.

Kind regards,

Nitish Gogoi, Ph.D.

Academic Editor

PLOS One

Additional Editor Comments (optional):

Reviewers' comments:

# Editor report: Acceptance letter

**DOI:** 10.1371/journal.pone.0361592.r004

**Authors:** Nitish Gogoi

PONE-D-26-31876R1

PLOS One

Dear Dr. Chiu,

I'm pleased to inform you that your manuscript has been deemed suitable for publication in PLOS One. Congratulations! Your manuscript is now being handed over to our production team.

At this stage, our production department will prepare your paper for publication. This includes ensuring the following:

* All references, tables, and figures are properly cited

* All relevant supporting information is included in the manuscript submission,

* There are no issues that prevent the paper from being properly typeset

You will receive further instructions from the production team, including instructions on how to review your proof when it is ready. Please keep in mind that we are working through a large volume of accepted articles, so please give us a few days to review your paper and let you know the next and final steps.

Lastly, if your institution or institutions have a press office, please let them know about your upcoming paper now to help maximize its impact. If they'll be preparing press materials, please inform our press team within the next 48 hours. Your manuscript will remain under strict press embargo until 2 pm Eastern Time on the date of publication. For more information, please contact onepress@plos.org.

You will receive an invoice from PLOS for your publication fee after your manuscript has reached the completed accept phase. If you receive an email requesting payment before acceptance or for any other service, this may be a phishing scheme. Learn how to identify phishing emails and protect your accounts at https://explore.plos.org/phishing.

If we can help with anything else, please email us at customercare@plos.org.

Thank you for submitting your work to PLOS One and supporting open access.

Kind regards,

PLOS One Editorial Office Staff

on behalf of

Dr. Nitish Gogoi

Academic Editor

PLOS One
